# The pleiotropic effects of metformin: time for prospective studies

**DOI:** 10.1186/s12933-015-0273-5

**Published:** 2015-08-14

**Authors:** Daniel I Bromage, Derek M Yellon

**Affiliations:** The Hatter Cardiovascular Institute, 67 Chenies Mews, London, WC1E 6HX UK

**Keywords:** Metformin, Cardioprotection, Ischaemia–reperfusion injury, STEMI, Type 2 diabetes, PCI, Thrombolysis

## Abstract

The global prevalence of diabetes has risen to epidemic proportions and the trend is predicted to continue. The consequent burden of cardiovascular morbidity and mortality is a major public health concern and new treatments are required to mitigate the deleterious effects of cardiovascular disease in diabetic patients. Ischaemia–reperfusion injury is well known to exacerbate the harmful effects of acute myocardial infarction and subsequent therapeutic reperfusion, and several mechanical and pharmacological approaches to mitigating this injury have been investigated. Metformin, which is cheap, relatively safe and widely used in type 2 diabetes, is one such pharmacotherapy with considerable pre-clinical evidence for cardioprotective utility beyond its glucose-lowering effect. However, despite convincing basic evidence its translation to clinical application has largely been limited to studies of cardiovascular risk. There are several barriers to prospective randomized assessment in the context of acute myocardial infarction, not least the accessibility and already widespread use of metformin among patients with type 2 diabetes at high risk of cardiovascular events. In the place of class 1 evidence, well-designed prospective cohort studies of the potential pleiotropic utility of metformin in cardiovascular disease, and particularly its benefit in ischaemia–reperfusion injury, are needed. Given the availability of metformin worldwide, this is particularly true in low- and middle-income countries where the optimal therapy for acute myocardial infarction, primary percutaneous coronary intervention, may not be available, and instead patients are managed with thrombolysis. As this is less effective, metformin as an adjunct to thrombolysis (or PPCI) could represent an effective, cheap means of cardioprotection with global relevance.

## Background

The global prevalence of diabetes was estimated to be 9 % in 2014 and this looks set to rise dramatically [1, 2]. Cardiovascular disease accounts for 52 % of deaths among patients with type 2 diabetes [3], and therefore constitutes an important target for intervention. Of this excess cardiovascular mortality, an estimated 39 % is attributable to ST-segment elevation myocardial infarction (STEMI) [4]. Following STEMI, early reperfusion by primary percutaneous coronary intervention (PPCI) is the most effective strategy for reducing infarct size and improving clinical outcome [5, 6]. However, adverse sequelae persist: in a recent study, 30-day, 1-year, and 5-year all-cause (and cardiac) mortality rates following PPCI for STEMI were 7.9 % (7.3 %), 11.4 % (8.4 %), and 23.3 % (13.8 %), respectively [7]. It is known that patients with diabetes and coronary heart disease suffer worse clinical outcomes [8, 9], and in the aforementioned observational study, diabetes significantly increased the hazard of death over a median follow-up period of 4.7 years (fully adjusted HR 1.62 [95 % CI 1.32–1.97], p < 0.001).

A potential target in STEMI patients is the paradoxical injury inflicted by the therapeutic restoration of blood flow, known as ischaemia–reperfusion injury (IRI), which may exacerbate the final infarct size [10–13]. Several mechanical and pharmacological interventions have been investigated with respect to their ability to attenuate IRI [14], but to date no agent is in routine clinical use to protect the myocardium against IRI. Is there a role for metformin in this regard? Metformin is an oral antidiabetic agent of the biguanide class that exerts its effect by suppressing gluconeogenesis and increasing peripheral glucose uptake. However, the translation of metformin from a promising cardioprotective agent in the laboratory setting (see below) to implementation at the bedside has stalled, perhaps as a result of the widespread uptake of metformin as the first-line oral hypoglycaemic among patients with type 2 diabetes in high-income countries. Nonetheless, metformin is inexpensive and relatively widely available and its repurposing in STEMI could confer morbidity and mortality benefits both in the context of PPCI and in regions where PPCI is not readily available. To test this hypothesis, well-designed prospective cohort studies of the role of metformin in IRI are required.

## Basic evidence

**Fig. 1:**
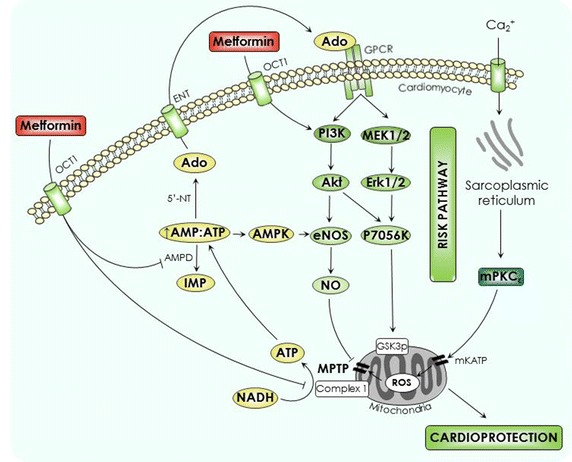
Proposed cardioprotective mechanisms of metformin. It is suggested that metformin confers cardioprotection by inhibiting mitochondrial complex I and inhibiting AMP deaminase, which both increase cytosolic AMP:ATP ratio. This activates AMPK causing the phosphorylation of eNOS, an integral part of the RISK pathway. Furthermore, increased AMP:ATP facilitates the extracellular diffusion of adenosine and its subsequent activation of the RISK pathway via a G protein-coupled receptor. Metformin may also activate PI3K directly. The RISK pathway inhibits MPTP opening which mitigates the detrimental effects of calcium influx and ROS generation at reperfusion. *Ado* adenosine, *AMP* adenosine monophosphate, *AMPK* adenosine monophosphate-activated protein kinase, *ATP* adenosine triphosphate, *eNOS* endothelial nitric oxide synthase, *ENT* equilibrative nucleoside transporter; Erk, extracellular signal-regulated kinases, *GPCR* G protein-coupled receptor, *IMP* inosine monophosphate, *MEK1/2* mitogen-activated protein kinase, *mK*_*ATP*_ mitochondrial ATP-sensitive potassium channel, *mPKC* mitochondrial protein kinase C, *MPTP* mitochondrial permeability transition pore, *NADH* nicotinamide adenine dinucleotide, *NO* nitric oxide, *OCT1* organic cation transporter 1, *PI3K* phosphoinositide 3 kinase, *RISK* reperfusion injury salvage kinase, *ROS* reactive oxygen species.

Several groups have demonstrated a significant reduction in infarct size in animal models of IRI following the administration of metformin [15–18]. It is hypothesized that the cardioprotective effect of metformin against IRI is independent of its hypoglycaemic actions [18]. We and others have investigated potential mechanisms for this phenomenon, which are thought to include activation of the RISK pathway either directly [15], by increased AMPK activation [17, 19], or via adenosine receptor stimulation [16], all of which inhibit mPTP opening at reperfusion and effect cardioprotection (see Fig. 1) [20, 21]. Interestingly, metformin has also been shown to reduce myocardial infarct size when administered 24 h and chronically prior to index ischaemia [18, 22].

## Clinical evidence

The UK Prospective Diabetes Study (UKPDS) was a large randomised, multicentre trial of glycaemic therapies in 5,102 patients with newly diagnosed type 2 diabetes [8]. It reported that metformin reduced the risk of AMI when compared to diet therapy alone in diabetic patients. Furthermore, metformin reduced all-cause and cardiovascular mortality compared to sulfonylureas and insulin, despite similar glycaemic control, which has been confirmed elsewhere [23–25]. Despite conflicting evidence from a large meta-analysis of randomized trials, which found no significant effect of metformin on the incidence of cardiovascular events versus active comparator [26], only a handful of studies have specifically evaluated the impact of metformin on IRI after STEMI. For example, Zhao et al. reported that pre-treatment with metformin was associated with a reduction of no-reflow, a phenomenon in which IRI is implicated, in patients with diabetes mellitus after PPCI for STEMI [27]. Importantly, Lexis et al. investigated the effect of pre-treatment with metformin on surrogate markers of AMI size in diabetic patients undergoing PPCI for STEMI in a single centre in the Netherlands [28]. They found metformin treatment to be an independent predictor of smaller MI size, albeit when compared to group on diet alone rather than alternative oral hypoglycaemics. A further study by the same group that administered metformin to patients with STEMI after reperfusion failed to find any improvement in left ventricular function, but here metformin was administered after the critical window of mPTP opening that is directly associated with IRI [29]. No study has yet evaluated the effect of pre-treatment with metformin on MACE endpoints after PPCI for STEMI.

## Missing evidence

Many of the clinical studies to date have been observational and/or use a placebo/no therapy comparator, which risks confounding by complex and poorly understood metabolic heterogeneity among patients with diabetes, including significant disparity in cardiovascular risk [30]. Furthermore, as mentioned, none have specifically examined the effect on mortality of metformin in the context of IRI, either among patients on metformin therapy at the time of STEMI or by administering metformin intravenously *at the time of* reperfusion (and therefore mPTP opening). This is likely due to the good availability and widespread uptake of metformin, at least in high-income countries, precluding a control group. Moreover, the potential randomised investigation of metformin in STEMI is hampered by the absence of licensed intravenous preparations and a lack of funding for investigation of a drug that is available off-patent. With this in mind, level 2 evidence may be the best that is realistically possible and as such there is a need for well-designed prospective cohort studies of the potential benefit of peri-STEMI metformin. Any such study should be adequately powered to account for the high number of confounding variables inherent in such a study. Finally, it is important to acknowledge the potential deleterious effects of nephrotoxicity and lactic acidosis attendant in the use of metformin in the context of PPCI for STEMI. It is hoped that prospective level 2 studies would clarify the risk–benefit balance of metformin use in this regard, however prior to any prospective randomised study a safety profile for this particular application would be essential.

## Conclusions

Type 2 diabetes and its cardiovascular sequelae represent a major global public health challenge. IRI is a major target for intervention in patients suffering STEMI, and may be of particular benefit in diabetic patients who have inflated cardiovascular mortality compared to non-diabetic patients. There is convincing pre-clinical evidence that metformin, given at the time of IRI, may have a cardioprotective role beyond its glucose-lowering effect. However, despite its availability and safety profile, there is a paucity of clinical studies addressing this hypothesis. To repurpose this potentially beneficial therapy to a global population in need of new treatments for STEMI, especially in regions where PPCI is unavailable, well-designed prospective cohort studies of the potential pleiotropic benefits of metformin on cardiovascular disease, and particularly its benefit in ischaemia–reperfusion injury, are essential.

## Abbreviations

CI: confidence interval
HR: hazard ratio
IRI: ischaemia-reperfusion injury
STEMI: ST-segment elevation myocardial infarction
PPCI: primary percutaneous coronary intervention
